# Supplementary material for: The Role of Primary Tumor Resection in Colorectal Cancer Patients with Asymptomatic, Synchronous, Unresectable Metastasis: A Multicenter Randomized Controlled Trial
Source: Cancers (Basel). 2020 Aug 16;12(8):2306. doi: 10.3390/cancers12082306 (PMC7464811; doi:10.3390/cancers12082306)
Supplement: Supplementary file 1 [file cancers-12-02306-s001.pdf]

cancers

MDPI

Article

The Role of Primary Tumor Resection in Colorectal Cancer Patients with Asymptomatic, Synchronous, Unresectable Metastasis: A Multicenter Randomized Controlled Trial

Eun Jung Park, Jeong-Heum Baek, Gyu-Seog Choi, Won Cheol Park, Chang Sik Yu, Sung-Bum Kang, Byung Soh Min, Jae Hwang Kim, Hyeong Rok Kim, Bong Hwa Lee, Jae Hwan Oh , Seung-Yong Jeong, Minkyu Jung, Joong Bae Ahn and Seung Hyuk Baik

Supplementary Materials

Table S1. Regimen of chemotherapy.

	**Arm 1 Upfront Chemotherapy Group (*n* = 22)**	**Arm 2 PTR Group (*n* = 26)**
1st line chemotherapy		
Regimen:		
FOLFOX	0 (0.0)	1 (3.8)
FOLFIRI	20 (90.9)	19 (73.1)
Capcitabine	1 (4.5)	1 (3.8)
XELOX	1 (4.5)	0 (0.0)
Target agents	–	–
Anti-VEGF	14 (63.6)	8 (30.8)
Anti-EGFR	5 (22.7)	9 (34.6)
2nd line chemotherapy		
Regimen:		
FOLFOX	10 (45.5)	14 (53.8)
FOLFIRI	2 (9.1)	2 (7.7)
Capcitabine	2 (9.1)	0 (0.0)
FL	1 (4.5)	0 (0.0)
Others	–	–
Target agents	–	–
Anti-VEGF	5 (22.7)	12 (46.2)
Anti-EGFR	0 (0.0)	0 (0.0)
3rd line chemotherapy		
Regimen		
FOLFOX	3 (13.6)	2 (7.7)
FOLFIRI	1 (4.5)	1 (3.8)
Capcitabine	2 (9.1)	2 (7.7)
XELOX	0 (0.0)	1 (3.8)
FL	1 (4.5)	0 (0.0)
Regorafenib	0 (0.0)	1 (3.8)
Mitomycin	0 (0.0)	1 (3.8)
Target agents	–	–
Anti-VEGF	1 (4.5)	1 (3.8)
Anti-EGFR	3 (13.6)	1 (3.8)

FL, 5-fluorouracil + leucovorin; VEGF. Vascular endothelial growth factor; EGFR, Epidermal growth factor receptor.
